# The Impact of Epitranscriptomics on Antiviral Innate Immunity

**DOI:** 10.3390/v14081666

**Published:** 2022-07-28

**Authors:** Beril Mersinoglu, Sara Cristinelli, Angela Ciuffi

**Affiliations:** Institute of Microbiology, Lausanne University Hospital, University of Lausanne, CH-1011 Lausanne, Switzerland; beril.mersinoglu@chuv.ch (B.M.); sara.cristinelli@chuv.ch (S.C.)

**Keywords:** epitranscriptomics, antiviral immunity, RIG-I sensing, IFN-I response, viral infection

## Abstract

Epitranscriptomics, i.e., chemical modifications of RNA molecules, has proven to be a new layer of modulation and regulation of protein expression, asking for the revisiting of some aspects of cellular biology. At the virological level, epitranscriptomics can thus directly impact the viral life cycle itself, acting on viral or cellular proteins promoting replication, or impacting the innate antiviral response of the host cell, the latter being the focus of the present review.

## 1. Introduction

Despite the central role of RNA in cellular biology, its highly degradable nature has complicated its study for years. Indeed, initially only a few RNA modifications were known and could be investigated, and most of them concerned ribosomal RNAs (rRNAs) or non-coding RNAs (ncRNAs), and later also transfer RNAs (tRNAs) and small nuclear RNAs (snRNAs). Although modifications of messenger RNA (mRNA) molecules such as polyadenylation (polyA) and 5′ capping have been known since the 1970s [1,2,3,4], the dynamic nature and role of other chemical RNA modifications have been underrated for a long time, owing mostly to the lack of sensitive technologies. Today’s tools and technologies facilitate the analysis of these numerous chemical modifications (more than 150 have been described to date, occurring at any position of any base) that are collectively termed the “epitranscriptome” [5,6,7,8,9,10]. During the last decade, mRNA modifications, consisting mostly of methylations, have been the focus of many investigations. Epitranscriptomic modifications of RNA molecules affect their molecular fate, largely impacting cell biology at multiple stages, including cancer biology [11], evolutionary studies [12], cell differentiation [13], autoimmune diseases [14], and infectious diseases [15]. The present review aims at providing a brief overview of epitranscriptomic modifications and their cellular role before focusing on their impact on viral replication, with particular attention paid to viral sensing and innate antiviral defense.

## 2. Epitranscriptomics

**Figure 1:**
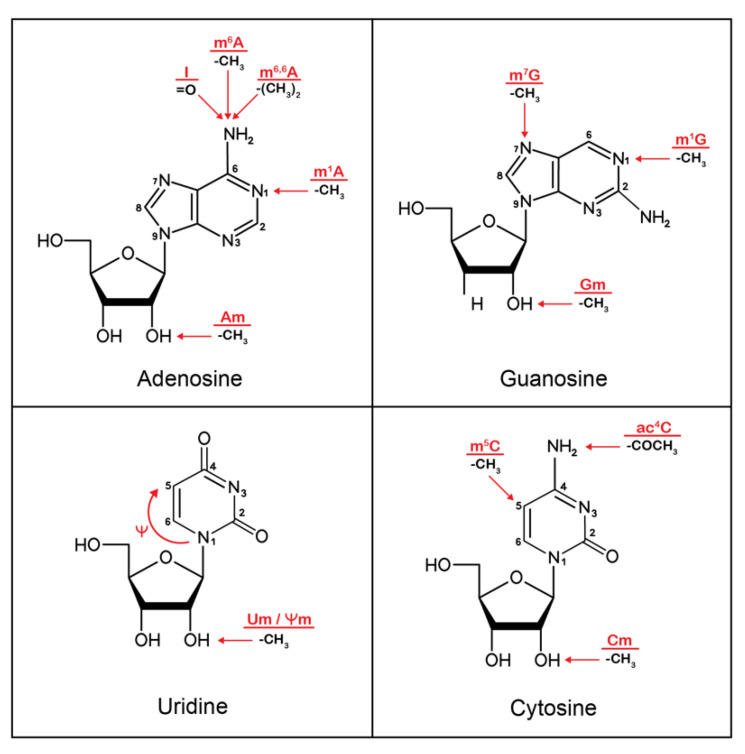
Major epitranscriptomic modifications. Each panel represents one of the four ribonucleotides. The precise locations of epitranscriptomic modifications predominantly found in mRNA are indicated with red arrows. The epitranscriptomic modifications are abbreviated in red, and the corresponding chemical group additions are indicated beneath. I: adenosine to inosine editing; m^6^A: N6-methyladenosine; m^6,6^A: N6-dimethyladenosine; m^1^A: N1-methyladenosine; Am: 2′-O-methylation of adenosine; m^7^G: N7-methylguanosine; m^1^G: N1-methylguanosine; Gm: 2′-O-methylation of guanosine; Ψ: pseudouridine; Um: 2′-O-methylation of uridine; Ψm: 2′-O-methylation of pseudouridine; m^5^C: 5-methylcytidine; ac4c: N4-acetylcytidine; Cm: 2′-O-methylation of cytosine.

Similar to DNA modifications, mRNA modifications are dynamic and can be reversible as they are co-transcriptionally added by specific “writer” enzymes (i.e., methyltransferases, acetylases) or removed by “eraser” enzymes (i.e., demethylases, deacetylases) [16]. Each chemical modification depends on its surrounding nucleotidic context and can be bound by specific proteins (“readers”), which will in turn impose RNA molecule fate, localization and function, such as nuclear retention [17], degradation [18,19] or translation efficiency [20], which eventually impacts cellular and viral processes. Beside 5′ capping and 3′ polyA modifications, a growing number of internal mRNA modifications have been described in the last decade [5,21,22]. Among them, N6-methyladenosine (m^6^A), pseudouridine (Ψ), Inosine (I) (through A-to-I editing), and 2′-O-ribose methylations (Nm) are the most extensively investigated (Figure 1). Although editing such as A-to-I (ADAR-mediated deamination of adenosine residues) and C-to-U (APOBEC-mediated deamination of C residues) are indeed *sensu stricto* RNA modifications, their role on cellular innate immunity has been extensively reviewed elsewhere (reviewed in [23,24,25,26]). This review will thus rather focus on the impact of the addition of chemical groups on innate cellular immunity.

In the past decade, epitranscriptomic studies on mRNA have been focusing extensively on m^6^A, i.e., the addition of a methyl group to the N6 position of adenosine (Figure 1), as it appears to be the predominant internal modification in mRNA [27,28,29]. M^6^A methylations are co-transcriptionally added by a large methyltransferase (MTase) complex composed of methyltransferase-like 3 (*METTL3*), methyltransferase-like 14 (METLL14), Wilms’ tumor 1- associating protein (WTAP) and other additional proteins [30,31], and can be removed by a demethylase, either AlkB homolog 5 (*ALKBH5*) or a fat mass and obesity-associated (FTO) protein [32,33]. The effect of m^6^A methylations on the RNA fate is mediated by the m^6^A binding proteins, also called “reader” proteins. The most widely studied m^6^A readers are YT521-B homolog domain family proteins (i.e., YTHDC1, YTHDC2, YTHDF1, YTHDF2, and YTHDF3); however, additional proteins can recognize m^6^A methylations, such as heterogeneous nuclear ribonucleoprotein (HNRNP) protein family members [34,35]. Although the redundant or distinct function of reader proteins is still controversial [36], it has been shown that YTHDF1 increases the translation efficiency of m^6^A-methylated mRNA [20], while YTHDF2 reduces the stability of m^6^A-methylated mRNA and mediate its degradation [18,37]. YTHDF3 functions together either with YTHDF1 and increases the translation efficiency of methylated mRNAs [38], or with YTHDF2 and enhances m^6^A-methylated mRNA decay [39].

Another abundant RNA modification is 2′-O-methylation of the ribose ring (Nm), which consists in the substitution of the 2′ hydroxyl of the ribose moiety of any RNA base with one methyl group (Figure 1) [40]. These 2′-O-methylations have been found on the first or first two transcribed nucleotides after the 5′-end cap structure (m^7^G cap0), and are referred to as cap1 and cap2, respectively [41]. 2′-O-methylation can also occur internally, in the coding region and near splice sites of mRNA [42,43]. These modifications are thought to be catalyzed by different sets of methyltransferases [41,44,45,46]; however, no eraser or reader proteins have been identified to date. The first transcribed adenosine can also be methylated at the N6 position, next to the 2′-O position, which forms an N6, 2′-O-dimethyladenosine (m^6^Am) modification [47,48]. The only identified methyltransferase catalyzing m^6^Am methylations is Phosphorylated CTD Interacting Factor 1 (PCIF1), which catalyzes only m^6^Am, but that does not affect m^6^A methylations [49,50]. The M^6^A eraser protein FTO selectively demethylates m^6^Am methylations [51]; however, proteins interacting with these methylations are yet to be identified. The other epitranscriptomic modifications, such as 5-methylcytidine (m^5^C) or N1-methyladenosine (m^1^A), are also starting to be explored and investigated [52,53,54,55,56].

Epitranscriptomic modifications regulate RNA-related cellular processes either in a direct manner, by affecting RNA secondary structures, or indirectly, by affecting access to or the efficiency of binding proteins [57,58,59]. M^6^A methylations have been shown to play an essential role in mRNA metabolism, including stability [60], nuclear export [17], splicing [61], translation [20], and mRNA decay [62]. The multiplicity of m^6^A-modulated molecular processes can in turn impact a large variety of physiological processes, such as cell differentiation [63], circadian rhythms [64], embryogenesis and fertility [65], tumorigenicity and cancer [11], neural development [13], and immunity [14]. Similarly, 2′-O-methylations of mRNAs have been linked to RNA stability [66], splicing [67], and translation [68], thereby impacting different physiological and pathological contexts, including innate immunity [69,70] and cancer [71,72]. M^6^Am methylations are poorly understood. They have only been shown to date to potentially affect antiviral immune response [73], mRNA stability [50,51] and translation efficiency [49], although the latter is still debated.

## 3. Epitranscriptomics and Innate Immunity

**Figure 2:**
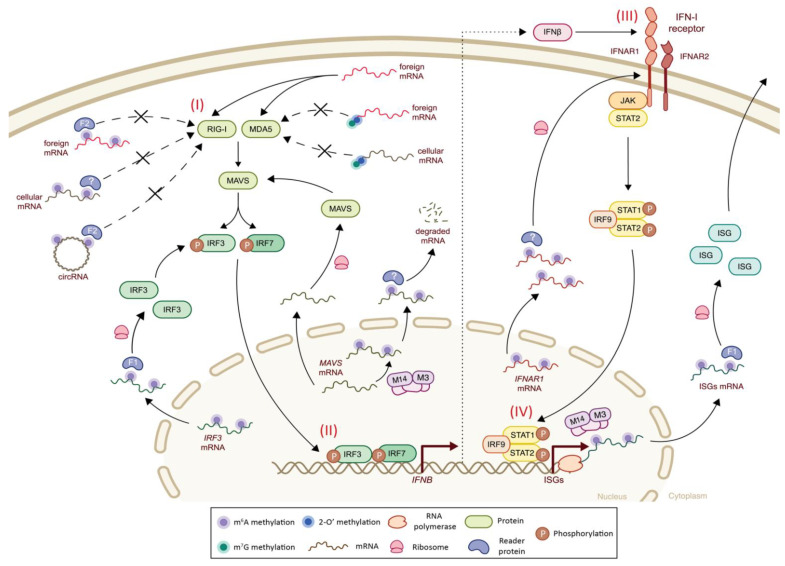
The impact of mRNA methylations in the innate immune response. (I) Foreign unmodified mRNAs (red), but not methylated mRNAs, are sensed in the cytoplasm through RLRs, RIG-I and MDA5, thereby triggering their activation and conformational changes to recruit MAVS adaptor protein, which in turn will activate TBK1 and IKKε (not shown), to induce IRF3 and *IRF7* phosphorylation. (II) Phosphorylated IRF3 and *IRF7* translocate to the nucleus and initiate *IFNB* expression. (III) IFNβ binds to the IFNAR receptor and activates JAK-mediated STAT2 phosphorylation, which will then bind to phosphorylated STAT1 and IRF9. (IV) The STAT1–STAT2–IRF9 complex translocates to the nucleus and triggers the expression of a variety of ISGs. RNA methylations are involved in many steps of this pathway, regulating the innate response through several mechanisms. Foreign and cellular mRNAs (grey) with a 5′-cap structure (m^7^G and 2′-O methylations, filled blue and green circles, respectively) do not trigger MDA5 sensing. Similarly, foreign and cellular mRNAs, and circRNAs with m^6^A-methylated residues (filled purple circle), interact with the YTHDF2 reader protein (F2, light blue), sensed as “self” and do not trigger RIG-I sensing. M^6^A methylations also regulate the translation of *IRF3*, *IFNAR1* and ISGs by increasing the translation efficiency of IRF3 and ISGs, via YTHDF1 (F1, light blue) binding, and by improving the mRNA stability of *IFNAR1*. M^6^A methylations also regulate MAVS protein levels by increasing the mRNA decay of methylated *MAVS* mRNA. F1: YTHDF1; F2: YTHDF2; M3: *METTL3*; M14: METTL14.

The innate immune response is the primary cell barrier to counteract pathogen invasion. To achieve this goal, the cell needs first to sense the invading pathogen and then to launch a response to prevent its replication or to induce cell suicide [74]. The first recognition of foreign genomes is mainly carried out by cellular sensors, which are pattern-recognition receptors and include endosomal Toll-like receptors (TLR), RIG-I like receptors (RLR), and NOD-like receptors (NLR), as well as non-receptor cytosolic sensors (Figure 2I) [75]. Of particular importance for viral genomic RNA sensing are retinoic acid-inducible gene-I (RIG-I) and melanoma differentiation-associated protein 5 (MDA5), which can bind to different forms of viral RNA (single-stranded (ss) RNA, and short or long double-stranded (ds) RNA), undergo ATP-dependent conformational changes, and initiate signaling cascades [76,77,78]. This consists first in their interaction with mitochondrial antiviral-signaling (MAVS) adaptor proteins, which subsequently activates TANK-binding kinase 1 (TBK1) and IκB kinase-ε (IKKε), which in turn phosphorylates transcription factors, interferon-regulatory factors (IRF) 3 and 7, as well as nuclear factor-κB (NF-κB). Phosphorylation of these transcription factors leads to their nuclear translocation (Figure 2II), and type I interferon (IFN-I) expression, including IFNα, IFNβ and the less well-described IFNε, IFNτ, IFNκ, IFNω, IFNδ and IFNζ [79]. IFN-I is at the center of antiviral activity and acts as a conductor regulating the antiviral response. Indeed, secreted IFNα and β bind to interferon receptor (IFNAR) and induce Janus tyrosine kinase (JAK) and signal transducers and activators of transduction (STAT) signaling pathways (Figure 2III), resulting in the activation of the expression of interferon-stimulated genes (ISGs) (Figure 2IV) [75,79]. ISGs are antiviral effectors that directly act on viral products, by inhibiting them or degrading them, and modulate the antiviral state of the cell (reviewed in [74,79]).

The growing awareness of the potential that epitranscriptomics has for transcript regulation requires further investigation into its role in the innate immune response, and sensing in particular. Previous work has revealed that RNA cap methylations are important in self/non-self RNA discrimination [80,81,82,83]. Indeed, while capped cellular mRNAs do not trigger immune responses, uncapped pathogenic RNAs are recognized by cellular sensors and activate the IFN-I response [69,84]. In particular, 2′-O methylation of the first transcribed nucleotide was shown to protect against RIG-I sensing [85], and the methylation of the first two transcribed nucleotides protects against MDA5 sensing [69,84]. On the other hand, sensing can also be manipulated as part of the antimicrobial response through the action of some ISG proteins [86]. Indeed, as an example, the IFN-induced protein with tetratricopeptide repeats 1 (IFIT1 or ISG56) can recognize and directly bind to non-2′-O-methylated cap structures or uncapped 5′-ppp structures, thereby suppressing translation initiation (reviewed in [87,88]).

Similar questions were raised for m^6^A methylations, and studies have revealed that m^6^A methylations do indeed have the ability to modulate sensing efficacy or the innate immune signaling cascade (Figure 2) [89]. For example, cells may use m^6^A epitranscriptomic marks to differentiate self RNA from non-self RNA, thereby impacting the efficiency of cellular sensors (TLRs or RLRs) [84,90]. In other terms, while foreign or cellular non-methylated RNA (ssRNA and dsRNA) or circRNA efficiently activate the RIG-I signaling pathway, m^6^A-methylated RNA does not. Indeed, Durbin and colleagues have revealed that the sole addition of m^6^A to one specific RNA was able to trigger YTHDF2 reader binding, thereby reducing RIG-I binding, preventing RIG-I conformational change and failing to activate the signaling cascade, leading to *IFNB* transcription [84,91]. The exact mechanism by which YTHDF2 suppresses RIG-I conformational changes, and the possible implication for other factors, remain to be solved. This phenomenon is not restricted to the m^6^A mark as other epitranscriptomic modifications, including m^5^C and pseudouridine, seem to be able suppress RIG-I signaling as efficiently at different steps [84], suggesting that cellular sensors may recognize several RNA modifications in parallel, and that each may have a distinct mechanism for controlling “self” recognition. Finally, to ensure “self” recognition and avoid autoimmune responses, m^6^A methylations show additional functions, as they prevent not only the triggering of RIG-I sensing, but also the formation of cellular dsRNAs that would trigger RIG-I and MDA5-mediated IFN-I responses [92,93].

M^6^A residues can modulate the half-life of methylated mRNAs, thereby providing a fast alternative method of translational regulation that could be exploited by the host cell to regulate the immune response to external pathogens [89]. In vitro and in vivo studies have shown that *Mavs* mRNA is m^6^A methylated by METTL14 during the resting cellular state, which impairs its stability and induces RNA degradation, resulting in an attenuated IFN-I response [94]. In *Mettl14*-deficient mice, *Mavs* mRNA lacking m^6^A methylations showed improved stability, and hence, increased translation. This mechanism offers an m^6^A-driven negative feedback regulation upon overactivation of the IFN-I response, with possible involvement of other epitranscriptomic modifications. In most cases, however, m^6^A methylations favor positive regulation by enhancing the translation of methylated mRNAs. Indeed, the m^6^A writer *METTL3* was found to be phosphorylated by TBK1 on position S67, enhancing the interactions of *METTL3* with the translational complex and promoting mRNA translation [95]. Moreover, m^6^A methylations of *IRF3*, *IFNAR-1* and *ISGs* mRNAs lead to enhanced translation, either by increasing mRNA stability or by increasing mRNA translation [95,96,97]. Interestingly, 12 out of 14 ISG mRNAs associated with antiviral functions [98] were shown to be m^6^A methylated upon IFN-I signaling activation, leading to increased translation via YTHDF1 [97], thereby providing an additional example of the epitranscriptomic-associated mechanism of regulation.

## 4. Viral Epitranscriptomics and Innate Immunity

As viruses need host cells to replicate, it seems likely that they might be affected by chemical modifications as well. Hence, since 2015, multiple studies have explored and investigated the role of epitranscriptomic modifications during viral infection. These studies pointed out that (i) genomic viral RNA can be modified, and that these modifications have a significant impact on their viral life cycle; (ii) viral transcripts can be methylated by the cellular machinery, and (iii) the general epitranscriptomic landscape of the cell changes rapidly during infection, either to promote viral replication by virus-mediated hijacking, or to respond to the invading pathogen and prevent its replication through innate defense mechanisms [59,83,99]. These observations paved the way for further investigations into the possible roles of epitranscriptomic modifications in tuning the antiviral response, which is the focus of the present work (Table 1). Although viruses can induce both type I and type II interferon responses [100], only the type I interferon pathway has been shown to date to be affected by virus-modulated epitranscriptomic modifications. Depending on their replication class (according to Baltimore classification) and family, viruses exploit the cellular epitranscriptomic machinery to escape the innate immune response in different ways. Because RNA viruses may be logically more affected by RNA methylations, possibly occurring on both transcripts and the genome, we will focus on RNA viral replication classes first, and then on DNA viral replication classes.

## 5. RNA Viruses

### 5.1. Class III Viruses: Double-Stranded RNA

Double-stranded RNA (dsRNA) viruses constitute a diverse and heterogenous group of non-enveloped viruses, sharing similar strategies for replication, translation or defeating antiviral responses, thereby suggesting a common ancestry [117]. Upon entry of the host cell, genomic dsRNA segments are transcribed, generating positive-stranded viral ssRNA that are subsequently used as a template, either for protein translation, or for genome replication [118].

Rotaviruses (RV) belong to the *Reoviridae* family and hold 11 dsRNA genomic segments that are surrounded by three concentric capsid layers, without an envelope [119]. After cell entry, the outer capsid is removed, leaving a double-layered particle that is transcriptionally active [120] and that protects the dsRNA from the host cell innate immune recognition and degradation [121]. Indeed, dsRNA molecules are known so far to be the perfect substrate for RIG-I and MDA-5 recognition, although the impact of epitranscriptomic modifications on this dsRNA detection has yet to be redefined. Nonetheless, having dsRNA genomes inaccessible to the detectors through their enclosure within the double-layered particles allows viruses to escape intracellular detection, and continue their life cycle. The dsRNA molecules are then used as templates for transcription by the viral RNA-dependent RNA polymerase (RdRp) and production of viral mRNAs that are subsequently 5′capped by the activity of viral protein 3 (VP3), although with some variability [122,123]. Indeed, it was demonstrated that during the capping process, some viral mRNAs were not perfectly capped, and consequently, some viral mRNA molecules were left uncapped or partially capped with only m^7^G, but not 2′-O methylations, both allowing the triggering of RIG-I and MDA5 sensing, leading to an IFN-I response [121]. To circumvent viral RNA-mediated activation of the IFN signaling cascade, two RV proteins can lead to the proteasomal degradation of cellular proteins of the innate immune response: MAVS degradation is mediated by the viral VP3, while IRF3, IRF5 and *IRF7* degradation are mediated by the viral non-structural protein 1 (NSP1) [124,125].

Epitranscriptomic studies revealed that RV mRNAs (which are non-poly-adenylated transcripts) are m^6^A-methylated, and that the non-structural protein 3 (NSP3) mRNA displayed the highest number of methylations [93]. Interestingly, the viral NSP3 protein is highly involved in promoting viral protein translation, while shutting off cellular translation through the impairment of eIF2 and polyA-binding protein (PABP)—unlike cellular mRNAs, viral mRNAs are not poly-adenylated, and thus, do not rely on PABP for translation [126,127]. Nevertheless, the exact positions of viral m^6^A methylations and their exact role in RV replication remain to be elucidated.

**Figure 3:**
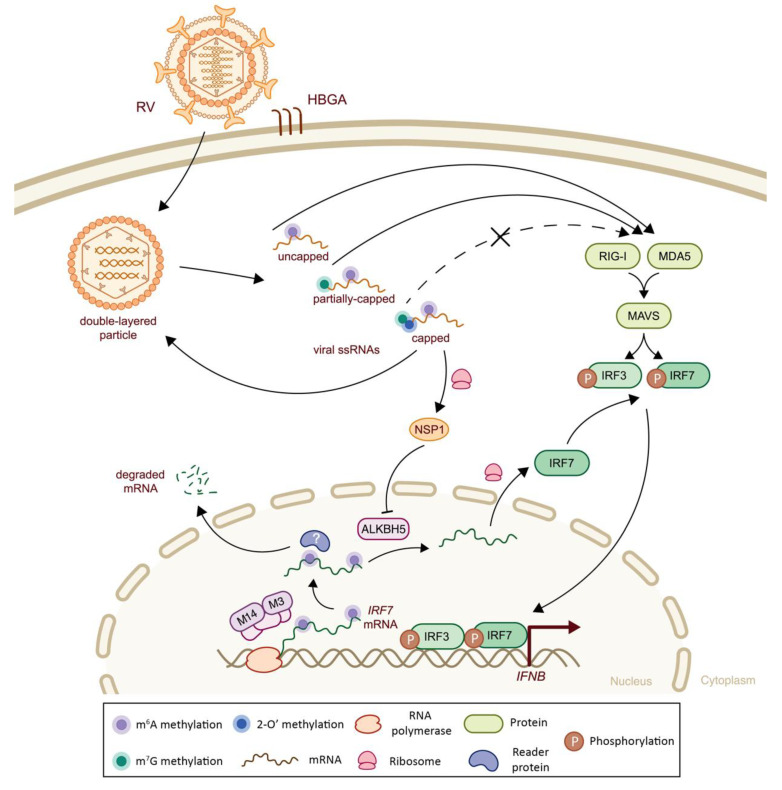
The impact of epitranscriptomic methylations on IFN-I response during RV infection. Upon genomic dsRNA release in the infected cell, rotaviruses (RV) produce non-poly-adenylated ssRNAs and use them as a template for translation and genome replication. RV ssRNAs (or mRNAs) are capped at the 5′ end by the guanylyltransferase and methyltransferase activities of the viral VP3 protein (not shown). However, the insufficient capping efficiency results in a mixed population of uncapped ssRNAs, partially capped ssRNAs harboring m^7^G methylations (filled green circles), or completely capped ssRNAs with both m^7^G and 2′-O methylations (filled blue and green circles, respectively). The 5′ capping profile dictates ssRNAs sensing, as uncapped and partially capped ssRNAs trigger RIG-I- and MDA5-mediated IFN-I responses, while completely capped ssRNAs escape from sensing. Additionally, viral mRNAs are also m^6^A-methylated (filled purple circles). During RV infection, the viral NSP1 protein reduces *ALKBH5* protein expression, resulting in the global increase in the m^6^A methylation of cellular transcripts, including *IRF7*. The increase in *IRF7* mRNA methylations results in decreased mRNA stability, hence enhanced degradation, reduced *IRF7* expression and an impaired IFN-I response. M3: *METTL3*; M14: METTL14.

RV can also alter the m^6^A methylation of host mRNAs, thereby modulating their responses to infection. Indeed, in vivo infection studies using a mouse model manifested a global increase on cellular m^6^A levels linked to decreased *Alkbh5* mRNA and protein levels, which is mediated by viral NSP1 protein activity (Figure 3). NSP1 prevents IFN-I response through the induced proteasomal degradation of many players, including IRF3 and *IRF7* [126,128]. Whether *ALKBH5* is another target of NSP1-mediated degradation or whether NSP1 is involved in additional strategies to evade innate immunity remains a question to be answered. Furthermore, the NSP1-induced decrease in *ALKBH5* protein level resulted in an impaired IFN-I response through its action upon *Irf7* mRNA. Indeed, transcriptomic analysis comparing wild-type and *Mettl3*-deficient mice revealed a negative association between m^6^A methylation and IFN-I response, i.e., the inhibition of m^6^A methylations correlated with elevated IFN-I response and related pathways. Further m^6^A profiling revealed the *Irf7* transcript was m^6^A methylated, with a peak near 3′UTR, and that this methylation lowered its stability, thereby impairing IFN-I induction. This mechanism was supported by the higher levels of *Irf7* mRNA observed in *Mettl3*-deficient mice compared to wild-type mice, which were correlated with the reduced viral replication observed in *Mettl3*-deficient mice, and in in vitro human cell line models [93]. The additional cellular factors that may be influenced by RV-mediated m^6^A methylation, as well as the mechanism of m^6^A-driven *IRF7* transcript degradation, remain to be clarified.

### 5.2. Class IV Viruses: Positive Sense, Single-Stranded RNA

The main characteristic of single-stranded positive sense RNA (ssRNA (+)) viruses is that, upon cell entry, their genomic RNA can be used directly as mRNA, and thus as a template for protein translation by the host ribosomes [129,130]. This class includes *Flaviviridae*, *Coronaviridae* and *Enteroviridae*, among others. Epitranscriptomic studies on these three families showed that their viral RNA is methylated, and that viral infection triggers changes at the host epitranscriptomic level [131,132]. Although changes in epitranscriptomic levels influence the host immune response, each viral family employs a unique mechanism to counteract the host immune response.

*Flaviviridae* encodes the NS5 protein, which has a methyltransferase activity capable of capping viral RNA with both N7 and 2′-O methylations [133,134]. Studies on Hepatitis C virus (HCV), Dengue virus (DENV), Zika virus (ZKV), West Nile virus (WNV), and Japanese encephalitis virus (JEV) showed that these methylations allow the viral genome to be camouflaged from cellular sensing radars and evade cell defense. An in vitro study on DENV carrying a mutated NS5 protein, i.e., with a functionally inactivated 2′O-MTase activity, showed the earlier induction of antiviral response compared to the wild-type virus, and hence, reduced viral replication during early infection stages [135]. Consistent with these observations, in vivo studies showed that mice challenged with NS5-mutated viruses (DENV, WNV and JEV) showed increased survival rates compared to mice challenged with the wild-type viruses [136,137,138,139]. NS5 showed additional IFN-I antagonizing actions, including suppressing ISG translation or inhibiting phosphorylation of different players of the IFN-I signaling cascade (reviewed in [140,141]). Interestingly, DENV and WNV NS5 can also catalyze 2′-O methylations of internal adenosines (Am) of viral transcripts and cellular ribosomal RNA, although with a lower frequency than cap methylations [142], which may suggest a putative role for NS5 in directly modulating 2′-O methylations of cellular transcripts, thereby potentially contributing to innate immunity escape.

**Figure 4:**
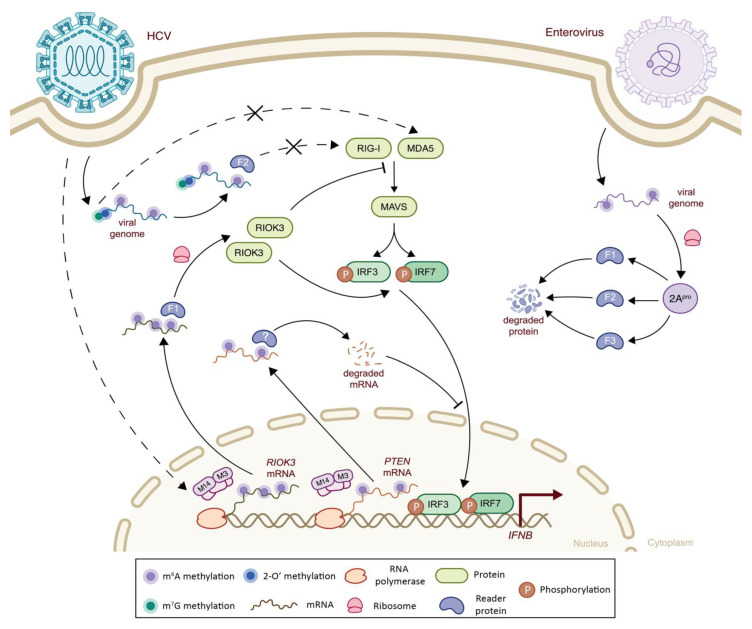
The impact of epitranscriptomic methylations on IFN-I response during HCV and Enterovirus infection. (*Left side*) Hepatitis C virus (HCV) genome carries a cap structure (m^7^G and 2′-O methylations at its 5′ end, filled blue and green circles, respectively), added by its own methyltransferase, NS5 (not shown), and is important to evade MDA5 sensing. M^6^A methylations (filled purple circles) of the HCV genome interact with the YTHDF2 (F2, light blue) protein and avoid RIG-I sensing. HCV infection also increases the m^6^A methylation levels of *RIOK3* and *PTEN* transcripts. The increase in m^6^A methylations of *RIOK3* increases its translation through YTHDF1 interaction and reduces HCV replication efficiency. *RIOK3* has a dual effect on the RLR-mediated antiviral signaling pathway: it phosphorylates MDA5 or induces RIG-I and MDA5 degradation, thereby inhibiting their activation on one hand, and acting as an adaptor protein for IRF3 phosphorylation, thereby promoting further signaling on the other hand. On the contrary, the increase in m^6^A methylations of *PTEN* induces mRNA decay, and hence lowers *PTEN* protein levels, which results in lower IRF3 nuclear translocation. (*Right side*) M^6^A methylations also present in Enterovirus genome. In the early stages of infection, 2A^pro^ viral protease cleaves YTHDF1-2-3 (F1, F2, and F3, respectively, light blue) reader proteins. The YTHDF3 protein has a positive regulatory effect on innate immunity by enhancing JAK/STAT signaling, and thus ISG production (not shown). F1: YTHDF1; F2: YTHDF2; F3: YTHDF3; M3: *METTL3*; M14: METTL14.

Although the global levels of m^6^A methylation on virion-associated RNA seem to be lower than the intracellular RNA, 19 m^6^A peaks were identified along HCV intracellular RNA molecules with an increase on the 3′ end [131]. The presence of m^6^A marks on HCV RNA molecules reduced the interaction between RIG-I and methylated RNA, thereby avoiding triggering the sensing cascade and evading the host antiviral response program (Figure 4) [105]. The mutation of the single m^6^A residue A8766C of HCV RNA, located 100 bp upstream of the PAMP recognition site, resulted in increased RIG-I sensing and increased IRF3 activation. Co-immunoprecipitation studies aiming at investigating interacting partners responsible for the decreased RIG-I sensing showed that YTHDF2 binds to methylated RNA at the RIG-I recognition site, thereby hindering its activation. As a control, YTHDF2 silencing was able to restore normal RIG-I sensing. Whether the reduced RIG-I sensing is correlated with a change in the stability of the YTHDF2-bound HCV genome or with a physical masking of the m^6^A-methylated genome remains an open question [37,105].

*Flaviviridae* members, in addition to altering their own RNA, are able to affect host m^6^A methylations to evade cellular immunity. HCV, for example, has been identified to trigger changes in the methylation profile of specific cellular transcripts, including Phosphatase and tensin homolog (*PTEN*). *PTEN* plays a prominent role in antiviral immunity by inducing IRF3 dephosphorylation and triggering its nuclear translocation, which favors the IFN-I cascade [143]. During HCV infection, increased m^6^A levels of *PTEN* mRNA have been reported to induce mRNA degradation, therefore contributing to a decreased IFN-I response [104]. Furthermore, the comparison of the m^6^A epitranscriptomic landscape of the host cell upon infection of one of four *Flaviviridae* (HCV, DENV, ZKV or WNV) with non-infected cells revealed that 51 genes were commonly differentially methylated and expressed during infection with all four viruses. These genes were enriched for innate immunity pathways, including NF-κB, TNF, and MAPK signaling. Quantification of m^6^A methylation changes during HCV, DENV or ZKV infection confirmed that 16 genes were m^6^A-hypermethylated and differentially expressed (although the normalization conditions were not specified). Increases in m^6^A levels, close to the stop codon in the 3′ UTR region, of one particular transcript drew more focus—Serine/Threonine-Protein Kinase RIO3 (RIOK3) [103,144], which is known to regulate the IFN-I dependent immune response in two opposite ways: (i) by hindering the IFN-I signaling via MDA5 phosphorylation [145] or via E3 ubiquitin ligase interaction, leading to RIG-I and MDA5 degradation [146], and (ii) by acting as an adaptor protein for TBK1 and IRF3 recruitment, thereby favoring their interaction [147]. Upon DENV, ZIKV and HCV infection, m^6^A-enriched *RIOK3* transcripts led to enhanced translation, which affects viral replication differently depending on RIOK3 activity in IFN-I signaling. Indeed, while the increase in *RIOK3* translation promoted DENV and ZIKV replication, it reduced HCV replication efficiency. The cause of this observed virus-dependent pro- or anti-viral effect of RIOK3 remains to be clarified [103].

*Coronaviridae* can also evade host defense mechanisms thanks to the presence of a cap structure on viral mRNA that is added through the action of virally encoded methyltransferases, the N7-methyltransferase NSP14 [148], and the 2′-O-methyltransferase NSP10-NSP16 complex [149,150]. Mutations of the NSP16 protein in severe acute respiratory syndrome coronavirus (SARS-CoV) (K46A, K170A, and D130A) and Middle East Respiratory Syndrome Coronavirus (MERS-CoV) (D130A) resulted in the suppression of their 2′-O-MTase activity and in the increased sensitivity to both IFN-I response and IFIT1 defense, thereby reducing in vivo pathogenesis without affecting MDA5 sensing-driven IFN-I expression [151,152]. To date, specific analyses showing internal ribose methylations, besides capping, catalyzed by the viral MTase complex have not yet been performed.

The COVID-19 pandemic situation drew global efforts into SARS-CoV-2 research. Several studies exploring its epitranscriptome confirmed that the viral genome and negative-sense RNA are m^6^A-methylated, with enrichments towards the 3′ end of the nucleocapsid (N) gene [101,153,154,155,156]. Investigation of epitranscriptomics role in innate immune sensing showed that the depletion of host m^6^A machinery (*METTL3*) led to the increased expression of immune response genes (i.e., pro-inflammatory cytokines *IL8*, *CXC1*, *CXCL3*, *CCL20*) in SARS-CoV-2-infected cells compared to non-infected controls. Furthermore, infection with m^6^A-depleted viral RNA resulted in a significant increase in viral RNA-RIG-I binding compared to a non-mutated virus, and SARS-CoV2 infection in cells with inactive *METTL3* resulted in enhanced IRF3 and IκBα phosphorylation, leading to an increase in downstream immune effector genes [101]. Interestingly, the comparison of *METTL3* expression profiles between patients affected by severe SARS-CoV-2 and non-infected patients showed decreased levels of *METTL3* and increased levels of ISGs, suggesting that reductions in m^6^A methylation may be correlated with higher inductions of immune response and cytokine storms [101,157,158].

Unlike *Flaviviridae* and *Coronaviridae*, enteroviruses (EV) lack a 5′ capping structure, which forces EV to use alternative strategies to evade innate immune sensing [159]. EV genomic RNA serves as an mRNA template for the production of a single polyprotein, which is then cleaved by two viral proteases, 2A (2A^pro^) and 3C (3C^pro^) [160,161]. These viral proteases are already known to impair the innate immune response by cleaving essential players of IFN-I signaling, i.e., RIG-I, MDA5, MAVS and *IRF7* [162,163,164]. More recently, 2A^pro^ was shown to cleave the m^6^A readers YTHDF1-3, inactivating them during the early phase of infection, and providing an alternative immune escape strategy (Figure 4). YTHDF3 is known to have a positive regulatory effect on JAK/STAT signaling and ISG induction, hence favoring the antiviral response; thus the viral-driven inactivation of YTHDF3 led to cascade downregulation. Even though the exact mechanisms of YTHDF3 proteolytic cleavage are yet to be described, this highlights another important role of m^6^A readers [102]. Although, m^6^A methylations were identified throughout the EV RNA [165,166], their impact on innate immunity, if any, has yet to be understood.

### 5.3. Class V Viruses: Negative Sense, Single-Stranded RNA

Epitranscriptomic changes can also have an impact on negative-sense, single-stranded RNA viruses (ssRNA (-)). Studies investigating the impact of epitranscriptomics in innate immune response have been carried out on several viral families with a non-segmented genome, such as *Pneumoviridae*, *Paramyxoviridae* and *Rhabdoviridae* [106,107,108], and a segmented genome, such as *Orthomyxoviridae* [167]. These viral families share similar replication, gene expression, and innate immune evasion mechanisms. Their negative-sense RNA genome is used as a template for positive-sense antigenomic RNA synthesis, which serves as an intermediate during replication in the cytoplasm [168]. However, they also display differences. For example, while viruses with non-segmented genomes synthesize 5′ capped viral mRNA via their own virally encoded capping enzyme [168,169], viruses with a segmented genome use a unique mechanism called “cap snatching”, where the 5′ cap structure of viral RNAs is stolen from cellular mRNAs by a virally encoded endonuclease [170,171].

The viral large (L) protein, one of the most conserved proteins in non-segmented *Mononegavirales*, contains four domains to catalyze viral replication and transcription (CR-I to -IV), and two domains to synthesize capping and cap methylation (CR-V and CR-VI) [172,173,174]. Analysis of the prototypic *Rhabdoviridae* vesicular stomatitis virus (VSV) has shown that the functional abrogation of m7G and 2′-O-MTase via introduction of three mutations in the MTase catalytic site of the L protein (K1651A, D1762A, and E1833Q) resulted in the production of viral particles with reduced pathogenicity. Comparative infection between mutated viral particles and non-mutated controls showed a decreased viral replication in vitro and impaired viral pathogenesis in an in vivo murine model [175,176]. However, defective virion-related decreased pathogenicity was not associated with reduced IFNβ levels, leaving the underlying mechanism to be clarified [176]. Interestingly, the first transcribed adenosine, adjacent to the capping structure of VSV mRNA, was identified as being m^6^Am-methylated [73]. However, this A nucleotide is not catalyzed by the L protein, but by the host phosphorylated C-terminal domain interacting factor 1 (PCIF1) methyltransferase, independently from viral m^7^G methylations, but dependently on 2′-O methylations. Although m^6^Am methylations of VSV mRNA do not appear to alter its stability or translation efficiency, the impact of m^6^Am methylations on mRNA’s stability and translation efficiency is still controversial and debated [49,50,51]. The catalytical inactivation of PCIF1 upon IFNβ treatment showed increased suppression of viral protein expression and further replication, suggesting the role of m^6^Am methylations in the evasion of host immune responses, in addition to 2′-O methylations [73]. The putative mechanism of m^6^Am hindering IFN-I responses and the extent of such a role of m^6^Am methylations on other viral mRNAs needs further confirmation.

**Figure 5:**
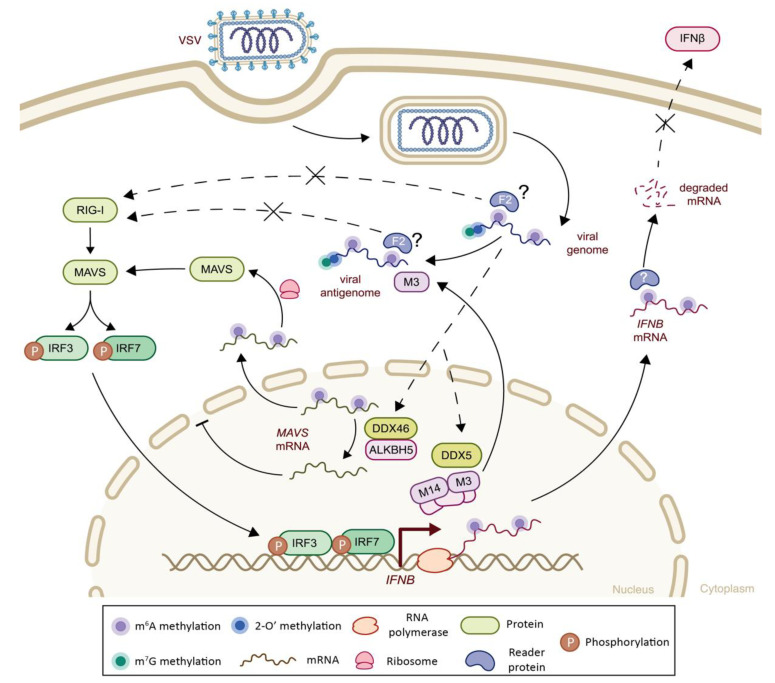
The impact of epitranscriptomic methylations on IFN-I response during VSV infection. Vesicular stomatitis virus (VSV) genome and antigenome carry a cap structure (m^7^G and 2′-O methylations at its 5′ end, filled blue and green circles, respectively), added by its own methyltransferase, L protein (not shown). M^6^A methylations (filled purple circles) of both the VSV genome and the antigenome inhibit RIG-I sensing and downstream IFN-I response, potentially through an interaction with YTHDF2 protein (F2, light blue). During VSV infection, *METTL3* (and possibly other components of the m^6^A writer complex) translocates to the cytoplasm to methylate newly transcribed viral mRNAs. Moreover, upon VSV infection, DDX46 recruits the ALKDH5 m^6^A eraser protein and demethylates *MAVS*, *TRAF3* (not shown) and *TRAF6* (not shown) mRNAs, thereby inhibiting their cytoplasmic translocation. Additionally, DDX5 interacts with *METTL3* and enhances the *METTL3*–METTL14 complex formation. Virally induced enhancement of the m^6^A machinery increases the m^6^A methylations of host transcripts including *IFNB, p65* (not shown), *IKKγ* (not shown), and *DHX58* (not shown). The increased m^6^A methylation of *IFNB* increases its mRNA degradation and hence IFNβ protein levels. F2: YTHDF2; M3: *METTL3*; M14: METTL14.

The epitranscriptomic analysis of ssRNA (−) viruses showed that all viral RNA molecules (genomic viral RNA, antigenomic RNA and viral transcripts) were m^6^A-methylated [107,108,177]. Influenza virus (IAV) belongs to the *Orthomyxoviridae* family and displays major m^6^A sites on four of the eight segmented viral genomes and transcripts, favoring the open reading frame (ORF) of viral structural proteins [178]. The IAV infection of m^6^A-deficient cells (generated by *METTL3* or YTHDF2 depletion) showed elevated levels of *IFNB* and reduced viral RNA levels compared to wild-type cells [109]. Among the *Pneumoviridae* family, m^6^A profiling of human metapneumovirus (HMPV) revealed 5 m^6^A peaks along the viral genome, 12 peaks in the antigenome and three m^6^A-methylated mRNAs out of eight viral mRNAs, mostly in overlapping regions [107]. Human respiratory syncytial virus (RSV), another member of the same viral family, presented 9 peaks in the viral genome, 15 peaks in the antigenome and 12 peaks in the viral transcripts [177]. In both viruses, m^6^A methylations were enriched in the envelope glycoprotein (G) region [107,177,179]. The presence of m^6^A methylations in both the HMPV and RSV viral genome and antigenome was shown to have a pro-viral effect in the viral life cycle (Figure 5). Indeed, the overexpression of m^6^A writers (*METTL3* and METTL14) or readers (YTHDF1-2-3) in host cells increased viral mRNA and protein levels, as well as viral particle release [108,177]. Moreover, m^6^A-methylated viral transcripts failed to trigger RIG-I sensing in vitro and in vivo in a rat model. On the contrary, m^6^A-deficient HMPV or RSV RNAs increased RIG-I binding affinity, triggered the conformational oligomerization of RIG-I, induced higher IRF3 phosphorylation, and hence enhanced the overall IFN-I induction [107,108,167]. Interestingly, microscopic analysis further revealed that the HMPV nucleocapsid (N) protein strongly colocalized with m^6^A writer METTL14 in the cytoplasm, suggesting that HMPV recruits the host m^6^A machinery to the viral replication site [107]. However, no significant colocalization has been detected between RSV N protein and m^6^A writer proteins [177]. Nonetheless, the direct interaction between HMPV N protein and METTL14 and the influence of the N protein on viral m^6^A methylations needs further investigation.

VSV and Sendai virus (SeV) from *Paramyxoviridae* also take advantage of m^6^A modifications to escape RIG-I-mediated innate immune recognition. In particular, 6 m^6^A peaks were detected in VSV genome, and 21 peaks and 6 peaks were detected in VSV and SeV antigenomes, respectively. Both viruses showed six similar m^6^A peaks along N and P viral transcripts. Similar to HMPV and RSV, m^6^A-deficient SeV and VSV virions induced higher IFN-I responses through increased RIG-I binding and increased IRF3 phosphorylation, through interaction with the YTHDF2 reader protein [107]. Microscopic analysis showed that VSV infection triggers the cytosolic translocation of *METTL3*, enabling viral access to m^6^A-machinery in order to methylate its own RNA and alter host RNA modifications. During VSV infection, the depletion of m^6^A machinery (*METTL3* or YTHDF2) showed increased levels of *IFNB* and *ISG15* mRNAs, suggesting that m^6^A methylations of *IFNB* mRNA led to reductions in IFNβ signaling. Moreover, the presence of m^6^A methylations induces the reshaping of the viral RNA, thereby impairing dsRNA formation and limiting RIG-I-mediated viral sensing. These observations were further validated *in vivo*, on tissue-specific *Mettl3*-deficient mice, and this confirmed that a reduction in m^6^A levels resulted in a more performant interferon response and a higher survival rate during VSV challenge [106].

Additional studies have revealed that DEAD-box (DDX) helicases act as negative regulators of antiviral response during VSV infection [110,111]. DDX46, which is normally involved in pre-spliceosome assembly, has been shown to bind transcripts involved in the interferon cascade (*MAVS*, *TRAF3* and *TRAF6*) and to interact with the m^6^A demethylase *ALKBH5*, in response to VSV infection in vitro and in in vivo murine model. In this way, DDX46 recruits *ALKBH5* and induces demethylation of *MAVS*, *TRAF3* and *TRAF6*, favoring their nuclear retention and consequently reducing their translation [111]. However, as mentioned previously, there are controversial data on the effect of m^6^A methylations of *MAVS* transcripts, and these need further clarification. Another DDX helicase, the DDX5 transcriptional activator, was shown to interact with *METTL3* in the nucleus and to enhance *METTL3*–METTL14 heterodimer formation in VSV-infected cells. This resulted in an increased m^6^A methylation, and consequently the enhanced nuclear export of two regulatory proteins of the NFκB cascade (namely p65 and IKKγ) and one regulator of RLR-mediated antiviral signaling (DExH-Box Helicase 58 (DHX58)). m^6^A methylations of *p65* and *IKKγ* favored their binding with YTHDF2 and resulted in their mRNA decay, while m^6^A methylations of DHX58 increased its translation and DHX58–TBK pathway activation [110]. Both those mechanisms contributed to a reduced antiviral response during VSV infection. Interestingly, the roles of DDX46 and DDX5 as negative innate immune regulators have been validated in the murine model [110,111]. These findings suggest that *METTL3* and DDX5 suppress innate immune responses by not only methylating viral RNA and hiding them from RLR sensing, but also directly affecting IFN-I levels. The mechanisms used by VSV to hijack *METTL3* and DDX proteins are yet to be uncovered. While the role of DDX5 has been shown with SeV as well [110], the second proposed mechanism has been only studied using VSV as a model, opening a question of whether these mechanisms are specific to VSV replication or are a more general response to viral infection.

### 5.4. Class VI Viruses: Positive Sense, Single-Stranded RNA with Reverse Transcriptase

Human immunodeficiency virus (HIV) is one of the most extensively investigated positive sense single-stranded RNA with reverse transcriptase viruses. HIV holds two copies of its own genome that encode essential proteins needed for replication, and accessory proteins important for pathogenesis and immune escape [180,181]. Once inside the cell, its RNA genome is reverse-transcribed into a linear double-stranded viral DNA and integrated into the host chromosome. After integration, the cellular machinery is hijacked, to ensure viral transcription and translation, followed by the production and release of new viral particles [182].

**Figure 6:**
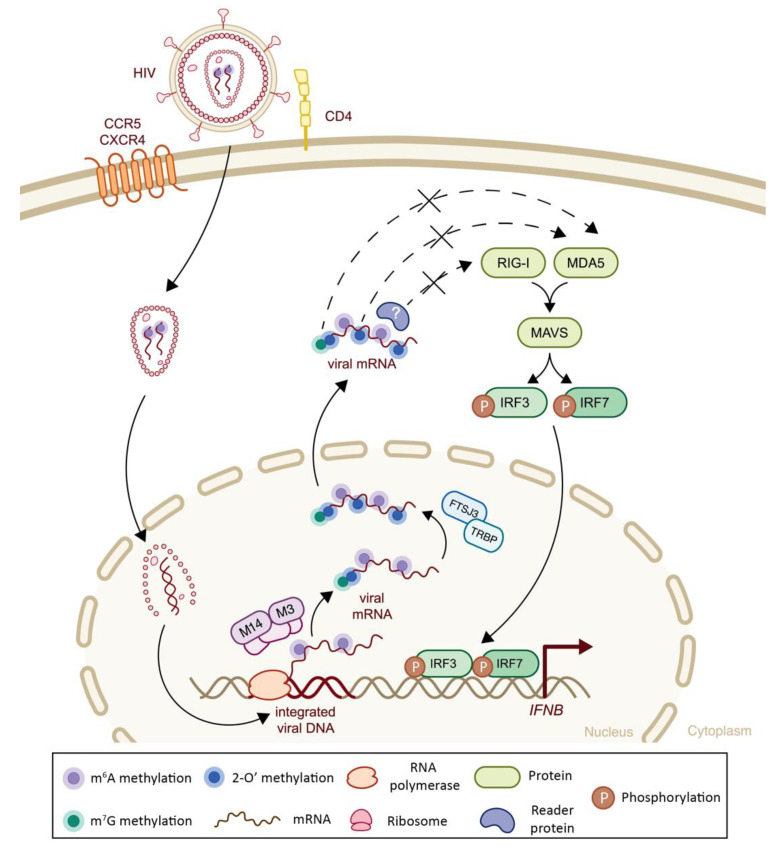
The impact of epitranscriptomic methylations on IFN-I response during HIV infection. After cellular entry, human immunodeficiency virus (HIV) reverse-transcribes its RNA genome into viral dsDNA and subsequently integrates into the host chromatin. The newly transcribed viral RNAs contain a complete cap structure (m^7^G and 2′-O methylations at its 5′ end, filled green and blue circles, respectively) that is catalyzed by virally hijacked host capping machinery (not shown). Viral transcripts also hold internal 2′-O methylations (filled blue circles) that are catalyzed by FTSJ3 2′-O-Mtase, which is previously recruited by the TAR binding protein TRBP. Both capping and internal 2′-O methylations are involved in impairing MDA5 sensing. HIV mRNA is also m^6^A-methylated (filled purple circles) by the cellular m^6^A machinery, resulting in RIG-I sensing and further IFNβ signaling impairment. M3: *METTL3*; M14: METTL14.

Although HIV does not encode its own 2′-O-Mtase [183], the viral Tat protein hijacks the host capping machinery in order to add the 5′-cap structure on its own RNA molecules [184]. In addition to a methylated cap, HIV mRNAs contain 17 internal 2′-O methylations, mostly on adenosine residues, added through the recruitment of a host 2′-O methyltransferase FtsJ RNA 2′-O-Methyltransferase 3 (FTSJ3) via the transactivation response element RNA-binding protein (TRBP) [43]. The presence of these 2′-O methylations on viral RNA allows for viral immune escape (Figure 6). Indeed, the infection of primary dendritic cells and macrophages with HIV strains lacking 2′-O methylations resulted in an increased IFN-I immune response compared to 2′-O methylated controls, which are able to avoid MDA-5 sensing, but not RIG-I recognition. Although the viral RNA genome should be hidden in the viral capsid from sensing, it is likely that some capsids are prematurely uncoated in the cytoplasm, rendering the genomic RNA accessible to innate immune detectors. These results demonstrate that HIV exploits RNA methylations to dodge innate immune response, and suggest that different epitranscriptomic marks may be recognized by different immune sensors.

The HIV RNA genome, as well as HIV transcripts, are also m^6^A-methylated and present a hotspot for m^6^A methylation toward the 3′ untranslated region (3′ UTR). According to several studies that investigated the epitranscriptomic marks of HIV and their impact on replication, m^6^A methylations could play both positive and negative roles on viral replication [54,185,186,187,188,189,190]. On one hand, m^6^A-enriched 3′ UTR bind to the YTHDF2 reader protein and thereby confer increased stability of viral mRNAs and increased gene expression [187]. On another hand, the Rev response-element (RRE) region is also m^6^A-methylated, promoting enhanced Rev binding, and hence, the enhanced nuclear export of Rev-bound transcripts [185]. Consistent with these observations, the activation of the writer complex (*METTL3*–METTL14–WTAP) by small-molecule activators caused a general increase in m^6^A nucleotides in the viral genome, resulting in enhanced translation and viral particle production [191]. Controversially, YTHDF1-3 proteins were shown to bind to two conserved m^6^A methylation motifs in the 5′UTR of the viral genome [192] and to inhibit its reverse transcription through RNA degradation, thereby inhibiting HIV replication [186,192]. Nevertheless, in most cases, the viral RNA genome is protected by the capsid throughout its voyage to the nucleus [193,194]; it is thus not clear at which stage and to what extent does the true impact of this observation play out on viral replication.

Despite these results, there has been very limited information on how these methylations impact HIV immune evasion. Chen et al. used m^6^A-deficient and m^6^A-methylated HIV RNA to show that m^6^A presence facilitated evasion from RIG-I protein sensing, and consequently the blocking of *IFNB* mRNA induction, in both the differentiated monocytic cell line and primary macrophages [112]. They further demonstrated that HIV RNA oligos containing only one single m^6^A methylation in the 5′ UTR region could significantly suppress IFNβ expression [112]. Given that HIV RNA carries different types of RNA methylations, including m^6^A [54], internal 2′-O methylations [43], m^5^C [188], m^7^G [195], m^1^A [188] and m^6^Am [196], as well as other modifications (reviewed in [197]), it is possible that they may all contribute to innate immunity evasion strategies, either individually or in combination. Finally, the RNA methylation landscape of host cells is modified upon HIV infection, displaying increased m^6^A-methylated host transcripts, and it may thus offer novel opportunities for HIV to exploit cellular factors and hide from innate immune responses [54].

## 6. DNA Viruses

### 6.1. Class VII Viruses: Double-Stranded DNA with Rverse Transcriptase

The Hepatitis B virus (HBV) carries a gapped relaxed double-stranded circular DNA (rcDNA) genome. After infection, the genomic rcDNA translocates to the host cell nucleus and is converted into a covalently closed circular DNA (cccDNA), a minichromosome that can persist and that serves as a template for transcription. RNA molecules that are produced comprise viral mRNAs aimed at translation and viral protein production, and pregenomic RNAs (pgRNA) aimed at reverse transcription for HBV DNA genome production [198,199]. As HBV transcription occurs in the host nucleus, it exploits the host capping mechanism [200]. Nevertheless, no data have been reported so far regarding the putative role of HBV capping in immune evasion.

**Figure 7:**
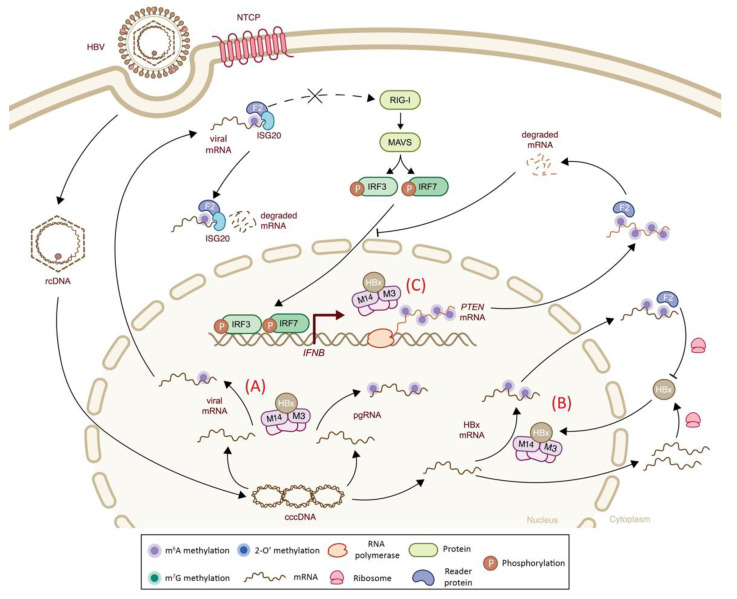
The impact of epitranscriptomic methylations on IFN-I response during HBV infection. Hepatitis B virus (HBV) carries a relaxed-circular DNA (rcDNA) genome that is converted into covalently closed circular DNA and transcribed into HBV RNAs upon host entry. (**A**) Viral mRNAs are co-transcriptionally m^6^A-methylated (filled purple circles) via cellular m^6^A machinery that is recruited by the viral HBx protein. M^6^A-methylated viral transcripts inhibit RIG-I sensing through m^6^A–YTHDF2 (F2, light blue) interaction. However, upon IFNα stimulation (not shown), YTHDF2 interaction with ISG20 leads to m^6^A-methylated viral mRNAs recognition and ISG20-mediated viral mRNA degradation. (**B**). HBx also alters its own methylation levels through its interactions with the m^6^A writer complex. While non-methylated *HBx* transcripts are translated into the HBx protein, m^6^A-methylated transcripts are degraded through the involvement of YTHDF2 protein. (**C**). HBx-mediated hijacking of host m^6^A machinery not only affects viral methylations, but also affects methylations of cellular transcripts, such as *PTEN*. Once *PTEN* mRNA is m^6^A-methylated, it is degraded through YTHDF2 recognition, and consequently, the decreased *PTEN* levels impair the nuclear import of IRF3. F2: YTHDF2; M3: *METTL3*; M14: METTL14.

The M^6^A profiling of HBV mRNA and pgRNA revealed one m^6^A methylation consensus motif on ε stem-loop (position A1907), located at the 3′ end of HBV mRNAs, and at both the 5′ and 3′ ends of HBV pgRNA, due to terminal redundancy [201]. An additional m^6^A site was identified at position A1616, within the coding region of the Hepatitis B virus X (HBx) transcript [115]. As the HBV ε RNA stem-loop was previously identified as an RIG-I recognition site [202], the presence of an m^6^A-methylated nucleotide at this location may alter RIG-I sensing and allow innate immune evasion (Figure 7). This hypothesis has been verified using m^6^A-mutated HBV, which was shown to boost RIG-I binding affinity to viral RNA and enhance IRF-3 activation, leading to higher IFN-I levels. Additionally, HBV m^6^A transcripts were shown to bind to YTHDF2, thereby disrupting RIG-I binding by competition and resulting in impaired IFNβ response [105]. The m^6^A-methylated A at position 1907 also plays a pivotal role in HBV mRNA decay as it can recruit the interferon-stimulated gene 20 (ISG20), a 3′→5′ exonuclease known to degrade HBV RNAs [114,203]. Indeed, the functional inactivation of m^6^A writers (*METTL3* or METTL14) or the YTHDF2 reader resulted in an increased HBV mRNA half-life, suggesting that m^6^A modification and YTHDF2 were both important for HBV mRNA stability [204]. Further studies revealed that upon IFNα treatment, ISG20 was expressed and bound to YTHDF2, independently from HBV. Upon HBV infection, the YTHDF2–ISG20 complex was then bound to the m^6^A1907 site on HBV mRNA and degraded thanks to its 3′→5′ exonuclease activity [114]. This observation may suggest that host cells counteract viral “hiding” attempts by “seeking” through YTHDF2. ISG20 exonuclease was reported to inhibit the replication of other viruses, including HCV, WNV, DENG and HIV [205,206,207,208]; whether ISG20-mediated viral degradation via m^6^A-YTHDF2 recognition is a mechanism unique to HBV or whether it is shared among different viral families remains to be investigated.

Interestingly, HBV encodes a protein, HBx, able to induce m^6^A methylations in both viral and host transcripts during infection. The HBx protein is an essential viral protein for viral replication, as it is a transcriptional trans-activator for both host and viral gene expression on the one hand [209,210], and suppresses RIG-I mediated signaling, hence suppressing the innate immunity of the host cell on the other [211]. Recently, HBx was also shown to interact directly with *METTL3*–METTL14 m^6^A writers, favoring their nuclear import and their recruitment at the transcription site, thereby promoting the co-transcriptional methylation of viral transcripts during the HBV life cycle. HBx is considered a virus-specific cofactor for m^6^A methyltransferase activity, as its depletion leads to the complete absence of m^6^A modifications along HBV RNAs [113]. In this way, HBx can also alter the m^6^A profile of specific host transcripts, such as tumor suppressor factor *PTEN* [104,113]. *PTEN* is involved in IFN-I signaling by inducing IRF-3 dephosphorylation and enabling its nuclear import [143]. The HBx-driven methylation of *PTEN* 3′ UTR mRNA reduces its stability through YTHDF2 protein interaction, and consequently reduces its protein expression, thereby resulting in impaired IRF-3 nuclear import and the disruption of the IFN-I signaling pathway [104]. Similarly, the m^6^A methylation site present on HBx ORF at position A1616 is responsible for a decreased *HBx* mRNA stability and thus lower protein levels, providing a negative feedback loop through methylated viral mRNA–YTHDF2 interaction [115]. The exact mechanism of the m^6^A-mediated autoregulation of HBx has yet to be investigated, but defects in this process might be involved in HBV chronic infection and pathogenesis [115].

### 6.2. Class I Viruses: Double-Stranded DNA

The replication of double-stranded DNA viruses involves various mechanisms and strategies. *Herpesviridae* and *Adenoviridae* replicate in the nucleus of the host cell and rely partially or entirely on host transcription and translation machineries to ensure successful replication [212]. In contrast, *Poxviridae* encode most, or all, of the necessary proteins required for their replication cycle, including RNA capping enzymes [212,213]. Indeed, the J3 protein, encoded by poxviruses, is a 2′-O methyltransferase that methylates the first transcribed nucleotide of viral RNA [214,215]. As shown previously for coronaviruses and flaviviruses, the K175R mutation of the J3 protein leads to the loss of its 2′-O-MTase activity, and results in increased sensitivity to IFN-I response as well as in IFIT interference both in vitro and in an in vivo murine model, thereby supporting a conserved role for 2′-O methylations in the immune evasion of viruses that replicate in the cytoplasm [86].

**Figure 8:**
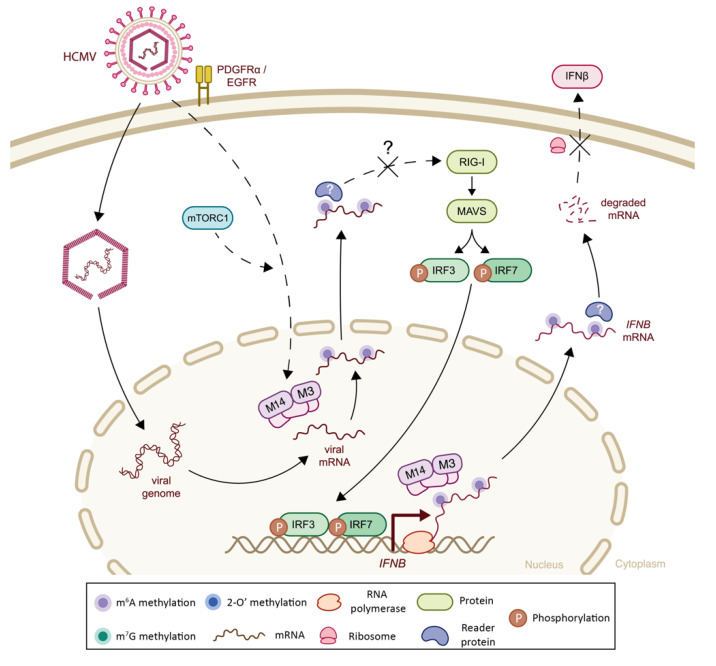
The impact of epitranscriptomic methylations on IFN-I response during HCMV infection. A double-stranded DNA genome of human cytomegalovirus (HCMV) is transcribed into viral mRNAs in the nucleus, after cellular entry. Viral transcripts are co-transcriptionally m^6^A-methylated (filled purple circles) by cellular m^6^A machinery. After HCMV infection, the m^6^A writer complex is upregulated, which is linked to HCMV-driven mTOR activation. M^6^A-methylated viral transcripts may avoid RIG-I sensing. HCMV infection also alters the *IFNB* m^6^A pattern. The m^6^A methylation of *IFNB* mRNA decreases its mRNA stability and thus IFNβ levels, supposedly via YTHDF2 interaction (F2, light blue). F1: YTHDF1; F2: YTHDF2; F3: YTHDF3; M3: *METTL3*; M14: METTL14.

Although, the majority of m^6^A epitranscriptomic profiling performed on dsDNA viruses have focused on Human Cytomegalovirus (HCMV), Epstein–Barr virus (EBV), Herpes Simplex virus type 1 (HSV-1), adenovirus and Kaposi’s sarcoma-associated herpesvirus (KHSV) [116,216,217,218], the influence of viral m^6^A methylations on innate immunity has only been explored in HCMV. Twenty-one m^6^A specific peaks have been identified in HCMV transcripts. These m^6^A modifications require a functional cellular m^6^A machinery and are necessary to achieve successful viral replication. The virus in turn influences the m^6^A methylation of host mRNAs to modulate their response during infection (Figure 8). Indeed, upon infection, the host m^6^A machinery, including writers (*METTL3*-METTL14), erasers (*ALKBH5*, FTO), and readers (YTHDC1, YTHDF1-3), was found to be upregulated at both the RNA and protein levels. Consistently, the silencing of m^6^A writers and readers resulted in a decreased HCMV replication efficiency, and m^6^A reader overexpression led to the opposite phenotype, i.e., increased replication [109,116]. To further dissect the interplay between HCMV and m^6^A, Rubio et al. investigated the impact of the mechanistic target of rapamycin (mTOR), as HCMV is known to modulate PI3K/AKT/mTOR signalling [116,219]. They showed that the pharmacological exposure of cells to the PP242 mTOR inhibitor resulted in the inhibition of the HCMV-induced increase in cellular m^6^A machinery players, suggesting that the increase in the virally induced m^6^A machinery was indeed dependent on HCMV-driven mTOR activation [116]. Interestingly, the HCMV UL38 early-expressed protein was previously shown to activate mTOR complex 1 (mTORC1) to regulate the cap-dependent translation of host transcripts in infected cells [220,221,222]. The UL38-mTORC1 pathway may thus also be involved in the activation of m^6^A machinery through the regulation of cap-dependent translation. Nevertheless, the direct impact of mTOR activation on m^6^A machinery remains to be clarified [116].

The genome-wide mapping of m^6^A methylations following HCMV infection showed that *IFNB* mRNA is enriched in m^6^A methylations with hotspots towards the coding sequence and the 3′ UTR [109,116]. Using site-directed mutagenesis, three m^6^A-methylated adenosine residues (A634, A684 and A689) located at the 3′ end of *IFNB* mRNA were identified as being involved in *IFNB* mRNA stability, showing that methylated *IFNB* transcripts resulted in lower transcript stability [109]. Consistently, depletion of the *METTL3* and METTL14 m^6^A methyltransferases increased *IFNB* mRNA stability, leading to ISG expression induction and decreased viral replication. In contrast, abrogation of the *ALKBH5* m^6^A demethylase was associated with the decreased accumulation of *IFNB* mRNA in the infected cell, thereby impairing cell-intrinsic antiviral immune response-related pathways and consequently allowing enhanced viral replication. The virally-induced m^6^A methylation of *IFNB* mRNA and the resulting impaired IFN-I response was also confirmed during Fowl Adenovirus Serotype 4 (FAdV-4) infection in vitro and in an in vivo murine model [116].

### 6.3. Class II Viruses: Single-Stranded DNA

The class of single-stranded viruses contains a wide range of viruses; no information on their epitranscriptomic landscape or on how they might use different methylations to escape cellular immunity has been reported yet.

## 7. Conclusions

Epitranscriptomic modifications represent a new layer of regulations for cell biology, as both the quantity and the quality of transcripts can impact their fate and function. Although current methodologies allow for exploring a few individual epitranscriptomic marks, further technological developments will be required to possibly reach comprehensive transcript characterization in the near future for in-depth understanding. As viruses co-evolve with their hosts, they have developed strategies to cope with epitranscriptomic modifications, in order to counteract and evade host innate immunity on one hand, and to promote their replication on the other hand. A better understanding of the viral mechanisms at play in these interactions likely provides novel opportunities for therapeutic interventions.

## Figures and Tables

**Table 1:** List of studies showing the impact of viruses on host immune response through epitranscriptomics.

**Class III: dsRNA Viruses**								
**Viral Family**	**Virus Name**	**Chemical Modification/** **Machinery**	**Analyzed Modified RNA**	**Detection Technique**	**Main Outcome(s)**	**Impact on Replication**	**Impact on Host** **Immune Response**	**Ref.**
*Reoviridae*	Rotavirus (RV)	m^6^A	Viral genome and host *IRF7* mRNA	m^6^A-seq^1^, m^6^A-qPCR^2^.	-Presence of m^6^A methylation on viral mRNAs, higher prevalence on *NSP3* mRNAs -Viral infection increases m^6^A methylation levels of host transcripts, by inhibiting *ALKBH5* protein expression via viral NSP1 protein, including *IRF7,* leading to lower mRNA stability, thereby impairing IFN-I signaling *Note:* In vivo *validation in murine model*	n.a.	(-) Decreased *IRF7* mRNA stability and subsequent IFN-I signaling	[93]
**Class IV: ssRNA (+) Viruses**								
**Viral Family**	**Virus Name**	**Chemical Modification/** **Machinery**	**Analyzed Modified RNA**	**Detection Technique**	**Main Outcome(s)**	**Impact on Replication**	**Impact on Host** **Immune Response**	**Ref.**
*Coronaviridae*	Severe Acute Respiratory Syndrome Coranavirus 2 (SARS-CoV-2)	m^6^A, m^6,6^A, 2′-O, ac^4^C, m^3^C, m^5^C, Ψ, m^5^U	Viral genome	LC-MS/MS-MS/MS/MS^3^ and m^6^A-seq	-Viral genome is highly methylated and the presence of m^6^A modifications on viral RNA impair RIG-I binding and consequently inhibit IFN-I signaling cascade -M^6^A methylations of host transcripts upon infection inhibit the expression of pro-inflammatory cytokines *Note: Severe COVID-19 patients correlate with a lower expression of m^6^A writers and higher induction of inflammatory genes*	+	(-) Decreased RIG-I sensing	[101]
*Picornaviridae*	Enterovirus (EV)	m^6^A machinery	n.a.	n.a.	-Enterovirus protease 2A cleaves YTHDF1-3 early in the infection phase, leading to suppression of the JAK/STAT signaling pathway	+	(-) Decreased JAK/STAT signaling	[102]
*Flaviviridae*	Dengue Virus (DENV)	m^6^A	Host *RIOK3* mRNA	m^6^A-seq	-Viral infection modulates m^6^A methylations of host transcripts involved in infection regulation, including *RIOK3* with increased m^6^A levels leading to induce translation and affecting IFN-I signaling	+	(-) Phosphorylation and inactivation of MDA5 sensor	[103]
*Flaviviridae*	Zika Virus (ZIKV)	m^6^A	Host *RIOK3* mRNA	m^6^A-seq	-Viral infection modulates m^6^A methylations of host transcripts involved in infection regulation, including *RIOK3* with increased m^6^A levels leading to induced translation and affecting IFN-I signaling	+	(-) Phosphorylation and inactivation of MDA5 sensor	[103]
	Hepatitis C Virus (HCV)	m^6^A	Host *RIOK3* mRNA	m^6^A-seq	-Viral infection modulates m^6^A methylations of host transcripts involved in infection regulation, including *RIOK3* with increased m^6^A levels leading to induced translation and affecting IFN-I signaling	-	(+) Increased TBK1-IRF3 interaction leading to increased IFN signaling	[103]
		m^6^A	Host *PTEN* mRNA	m^6^A-seq	-Virally induced m^6^A methylation of *PTEN* results in mRNA degradation via YTHDF2 binding, leading to cytoplasmic retention of IRF3 and inhibited IFN-I signaling pathway	+	(-) Decreased IRF3 nuclear import and subsequent IFN-I signaling	[104]
		m^6^A	Viral genome	n.a.	-Presence of m^6^A modification on viral RNA results in an impaired RIG-I sensing and a decreased IFN-I response via YTHDF2 binding	+	(-) Decreased RIG-I sensing	[105]
		m^6^A machinery	n.a.	n.a.	-*METTL3* acts as a negative regulator of the IFNβ innate immunity cascade in response to infection	n.a.	(-) Decreased IFNβ signaling	[106]
**Class V: ssRNA (−) Viruses**								
**Viral Family**	**Virus Name**	**Chemical Modification/** **Machinery**	**Analyzed Modified RNA**	**Detection Technique**	**Main Outcome(s)**	**Impact on Replication**	**Impact on Host** **Immune Response**	**Ref.**
*Paramyxoviridae*	Sendai Virus (SeV)	m^6^A machinery	n.a.	n.a.	-*METTL3* translocates to the cytoplasm and negatively regulates IFNβ innate immunity cascade in response to infection	n.a.	(-) Decreased IFN-I signaling	[106]
		m^6^A	Viral genome, antigenome and transcripts	m^6^A-seq	-Presence of m^6^A on viral RNAs impairs RIG-I activation and hinders IFN-I response	n.a.	(-) Decreased RIG-I sensing	[107]
*Pneumoviridae*	Human Metapneumovirus (HMPV)	m^6^A	Viral genome, antigenome and transcripts	m^6^A-seq	-m^6^A methylation of viral RNAs impairs RIG-I binding and the conformational change necessary to activate sensing and IFN-I response *Note:* In vivo *validation in murine model provided*	+	(-) Decreased RIG-I sensing	[108]
		m^6^A	Viral genome, antigenome and transcripts	m^6^A-seq	-Presence of m^6^A on viral RNAs impairs RIG-I activation and hinders IFN-I response	n.a.	(-) Decreased RIG-I sensing	[107]
	Human Respiratory Syncytial Virus (RSV)	m^6^A	Viral genome, antigenome and transcripts	m^6^A-seq	-Presence of m^6^A on viral RNAs impairs RIG-I activation and hinders IFN-I response	n.a.	(-) Decreased RIG-I sensing	[107]
*Orthomyxoviridae*	Influenza A virus (IAV)	m^6^A	Host *IFNB* mRNA	n.a.	-Viral infection induces m^6^A methylation of *IFNB* mRNA, leading to transcript destabilization and subsequent impairment of signaling cascade	+	(-) Decreased IFN-I signaling	[109]
*Rhabdoviridae*	Vesicular Stomatitis Virus (VSV)	m^6^A	Viral genome, antigenome and transcripts	m^6^A-seq	-Presence of m^6^A on viral RNAs impairs RIG-I activation and hinders IFN-I response	n.a.	(-) Decreased RIG-I sensing	[107]
		m^6^A	Host *IFNB* mRNA	n.a.	-m^6^A methylations of *IFNB* mRNA leading to transcript destabilization and subsequent impairment of signaling cascade	+	(-) Decreased IFN-I signaling	[109]
		m^6^A	Viral antigenome and transcripts	miCLIP-seq^4^ and m^6^A-qPCR	-*METTL3* translocates to the cytoplasm and promotes m^6^A modification on viral transcripts in response to infection, and negatively regulates IFNβ innate immunity cascade -Increase in m^6^A modifications reduces formation of viral dsRNA, thereby attenuating RLR sensing and IFN-I signaling cascade	+	(-) Decreased RLR sensing, decreased IFNβ signaling	[106]
*Rhabdoviridae*	Vesicular Stomatitis Virus (VSV)	m^6^A	Host *p65* and *IKKγ* mRNAs	m^6^A-qPCR	-Upon infection, DDX5 interacts with the *METTL3*–METTL14 complex promoting m^6^A modification of *p65* and *IKKγ* and their consequent degradation by YTHDF2, resulting in suppression of innate immune response *Note: In vivo validation in murine model provided*	+	(-) Decreased NF-κβ signaling pathway	[110]
		m^6^A	Host *TRAF3*, *TRAF6* and *MAVS* mRNAs	m^6^A-qPCR	-Upon infection, DDX46 recruits *ALKBH5* and induces demethylation of transcripts involved in antiviral signaling (*TRAF3*, *TRAF6* and *MAVS*), resulting in their nuclear retention, impaired translation and inhibition of IFN-I signaling	+	(-) Decreased IFN-I signaling	[111]
		m^6^Am	Viral transcripts	2D-TLC^5^	-The first transcribed adenosine of viral mRNAs is m^6^Am-methylated, leading to impaired IFN-I response	n.e.	(-) Decreased IFN-I signaling	[73]
**Class VI: ssRNA (+) RT Viruses**								
**Viral Family**	**Virus Name**	**Chemical Modification/** **Machinery**	**Analyzed Modified RNA**	**Detection Technique**	**Main Outcome(s)**	**Impact on Replication**	**Impact on Host** **Immune Response**	**Ref.**
*Retroviridae*	Human Immunodeficiency Virus (HIV)	2′-O	Viral genome	RiboMethSeq^6^	-Recruitment of FTSJ3 by TRBP to viral RNA leads to catalyzation of internal 2′O ribose methylations, which impair MDA5 sensing and IFN-I signaling cascade	+	(-) Decreased MDA5 sensing	[43]
		m^6^A	Viral transcripts	n.a.	-m^6^A methylation of viral RNA impairs RIG-I sensing and consequent IFN-I response	+	(-) Decreased RIG-I sensing	[112]
**Class III: dsRNA viruses**								
**Viral Family**	**Virus Name**	**Chemical Modification/** **Machinery**	**Analyzed Modified RNA**	**Detection Technique**	**Main Outcome(s)**	**Impact on Replication**	**Impact on Host** **Immune Response**	**Ref.**
*Hepadnaviridae*	Hepatitis B virus (HBV)	m^6^A	Viral transcripts	n.a.	-Presence of m^6^A modification on viral RNA results in an impaired RIG-I sensing and a decreased IFN-I response via YTHDF2 binding	+	(-) Decreased RIG-I sensing	[105]
*Hepadnaviridae*	Hepatitis B virus (HBV)	m^6^A	Host *PTEN* mRNA	m^6^A-seq	-Virally induced m^6^A methylation of *PTEN* results in mRNA degradation via YTHDF2 binding, leading to cytoplasmic retention of IRF3 and inhibited IFN-I signaling pathway	+	(-) Decreased IRF-3 nuclear import and subsequent IFNβ signaling	[104]
		m^6^A	Viral transcript and host *PTEN* transcript	m^6^A-qPCR	-Viral HBx protein recruits *METTL3*–METTL14 complex to catalyze m^6^A methylation of viral mRNAs and increases host *PTEN* mRNA methylation that alters IFN-I response	n.a.	(-) Indirect effect on IFN-I signaling	[113]
		m^6^A	Viral transcripts	n.a.	-Presence of viral m^6^A methylations at ISG20 binding position results in IFN-⍺-mediated viral mRNA degradation via ISG20-YTHDF2 complex	-	(+) Increased ISG activity	[114]
		m^6^A	Viral transcripts	n.a.	-m^6^A methylation of *HBx* transcript mediated by its own protein leads to decreased mRNA stability via YTHDF2 binding	-	n.a.	[115]
**Class I: dsDNA Viruses**								
**Viral Family**	**Virus Name**	**Chemical Modification/** **Machinery**	**Analyzed Modified RNA**	**Detection Technique**	**Main Outcome(s)**	**Impact on Replication**	**Impact on Host** **Immune Response**	**Ref.**
*Adenoviridae*	Fowl Adenovirus Serotype 4 (FAdV-4)	m^6^A	Host *IFNB* mRNA	m^6^A-seq	-Viral infection increases m^6^A methylation of IFN-I mRNA and leads to its destabilization	+	(-) Decreased IFN-I signaling	[109]
*Herpesviridae*	Human Cytomegalovirus (HCMV)	m^6^A	Host *IFNA* and *IFNB* mRNA	m^6^A-seq	-Viral infection increases m^6^A methylation of *IFNA* and *IFNB* mRNAs, leading to transcript destabilization and subsequent impairment of signaling cascade *Note:* In vivo *validation in murine model provided*	+	(-) Decreased IFN-I signaling	[109]
		m^6^A	Host *IFNB* mRNA	m^6^A-seq	-Viral infection increases the level of m^6^A machinery and induces *IFNB* mRNA methylation	+	(-) Decreased IFNβ signaling	[116]
*Herpesviridae*	Human Cytomegalovirus (HCMV)	m^6^A	n.a.	n.a.	-*METTL3* acts as a negative regulator of the IFNβ innate immunity cascade in response to infection	n.a.	(-) Decreased IFNβ signaling	[106]
	Herpes Simplex virus (HSV)	m^6^A	n.a.	n.a.	-*METTL3* translocates to the cytoplasm and negatively regulates IFNβ innate immunity cascade in response to infection	n.a.	(-) Decreased IFNβ signaling	[106]

^1^m^6^A-seq: N^6^-methyladenosine-sequencing, a technique based on immunoprecipitating RNA using m^6^A-specific antibodies, followed by high-throughput sequencing. ^2^m^6^A-qPCR: A technique based on immunoprecipitating RNA using m^6^A-specific antibodies, followed by reverse transcription and quantitative PCR. ^3^LC-MS/MS-MS/MS/MS: Liquid chromatography–tandem mass spectroscopy, a technique based on oligonucleotide separation and quantitative measurement of the modified ribonucleotides. ^4^miCLIP-seq: M^6^A individual nucleotide-resolution cross-linking and immunoprecipitation sequencing, a technique based on cross-linking m^6^A RNA to m^6^A-specific antibodies, followed by reverse transcription and high-throughput sequencing. ^5^2D-TLC: Two-dimensional thin-layer chromatography, a technique based on separating nucleotides and methylated nucleotides into two dimensions. ^6^RiboMethSeq: A technique based on alkaline fragmentation of methylated RNA, followed by high-throughput sequencing. n.a.: not applicable, n.e.: no effect.
